# Effects of substance misuse and family history of substance use disorder on brain structure in patients with attention-deficit/hyperactivity disorder and healthy controls

**DOI:** 10.1192/j.eurpsy.2021.366

**Published:** 2021-08-13

**Authors:** M. Novi, M. Paraskevopoulou, D. Van Rooij, A. Schene, J. Buitelaar, A. Schellekens

**Affiliations:** 1 Department Of Clinical And Experimental Medicine, University of Pisa, Pisa, Italy; 2 Department Of Psychiatry, Radboud University Medical Center, Nijmegen, Netherlands; 3 Department Of Cognitive Neuroscience, donders Institute For Brain, Cognition, And Behaviour, Radboud University Medical Center, Nijmegen, Netherlands; 4 Department Of Psychiatry, Donders Institute For Brain, Cognition And Behaviour, Radboud University Medical Center, Nijmegen, Netherlands

**Keywords:** Attention-deficit/hyperactivity disorder, Substance Use Disorder, Cortical thickness, Subcortical volumes

## Abstract

**Introduction:**

Literature shows overlapping alterations in brain structure in Attention-deficit/Hyperactivity Disorder (ADHD) and substance use disorder (SUD), suggesting shared pathophysiological mechanisms. It is unclear to what extent family history (trait) effects and/or substance misuse (state) effects explain the observed overlap.

**Objectives:**

Our aim was to examine the effects of (i) SUD family history (FH) and (ii) substance misuse on brain structure in ADHD.

**Methods:**

We compared structural MRI data (cortical thickness; subcortical volumes) between (i) ADHD subjects and controls with or without FH (ADHD-FH+: n=139; ADHD-FH-: n=86; controls-FH+: n=60; controls-FH-: n=74), and (ii) FH-matched ADHD groups with and without substance misuse and controls (ADHD+SM, ADHD-only and controls, n=68 per group). Furthermore, we explored whether FH effects were more pronounced in subjects with SUD in both parents (n=63) compared to subjects with one SUD parent (n=105) and without FH (n=160).

**Results:**

There was no main FH effect on brain structure. ADHD+SM showed decreased CT in inferior frontal gyrus (IFG) compared to controls, while no difference was found between ADHD-only and ADHD+SM or controls. Subjects with SUD in both parents showed decreased thickness of IFG and volume of nucleus accumbens (NAcc), compared to those with one SUD parent.

**Conclusions:**

Substance misuse in ADHD might result in smaller IFG, which is in line with findings in SUD-literature. A contribution of premorbid alterations, due to FH, could not be ruled out, particularly for IFG thickness. Future studies should further investigate the potential role of these regions in treatment and prevention strategies.

**Disclosure:**

No significant relationships.

